# Integrated Management of Co-Occurring Alcohol Use Disorder and Depression: Clinical Approaches for Concurrent Disorders

**DOI:** 10.1177/07067437251374564

**Published:** 2025-09-03

**Authors:** Anees Bahji, Victor Tang, Marlon Danilewitz

**Affiliations:** 1 Department of Psychiatry, 2129 University of Calgary, Calgary, Alberta, Canada; 2 Department of Psychiatry, 7938 University of Toronto, Toronto, Ontario, Canada; 3 Institute for Mental Health Policy Research, 25487 Centre for Addiction and Mental Health, Toronto, Ontario, Canada

**Keywords:** alcohol use disorder, major depressive disorder, concurrent disorders, integrated care, pharmacotherapy, psychotherapy, addiction psychiatry

## Abstract

Co-occurring alcohol use disorder (AUD) and major depressive disorder (MDD) are common and complex conditions that significantly impact patient outcomes. The bidirectional relationship between alcohol use and depression complicates diagnosis and treatment, as alcohol exacerbates depressive symptoms and vice versa. Integrated treatment addressing both disorders simultaneously has shown better outcomes compared to sequential treatments. This article provides evidence-based clinical guidance for managing patients with co-occurring AUD and MDD, focusing on pharmacotherapy, psychotherapy and integrated care models. Pharmacologically, selective serotonin reuptake inhibitors and tricyclic antidepressants are commonly used to treat depression in individuals with AUD, while naltrexone and acamprosate are first-line medications for AUD. Combining antidepressants with AUD medications improves treatment efficacy. Psychotherapeutic interventions such as Cognitive-Behavioural Therapy (CBT) and Motivational Interviewing are essential components of treatment, focusing on addressing both alcohol use and depressive symptoms. Behavioural activation has also proven effective in treating depression while reducing alcohol cravings. Integrated care models, where both disorders are addressed simultaneously, yield the best outcomes and involve coordinated pharmacotherapy, psychotherapy and ongoing follow-up care. A case example of a 33-year-old woman with AUD and MDD highlights the success of an integrated treatment approach, where a combination of sertraline, naltrexone and CBT led to significant improvements in both mood and alcohol use. Clinicians are advised to differentiate between alcohol-induced depression and primary MDD, consider potential medication interactions, and incorporate ongoing psychotherapy and monitoring for optimal patient outcomes. This approach emphasizes the importance of addressing both conditions concurrently to achieve better long-term recovery outcomes for patients with co-occurring AUD and MDD.

## Introduction

Co-occurring alcohol use disorder (AUD) and major depressive disorder (MDD) are prevalent and complex conditions in clinical practice.^1^ The bidirectional relationship between alcohol use and depression complicates diagnosis and treatment. Integrated care addressing both conditions is more effective than sequential treatment, yet often underutilized. This narrative review synthesizes current best evidence, including guideline-concordant care and recent meta-analyses, to provide clinician-oriented, practical guidance. It is not a systematic review or formal guideline, but offers an applied clinical framework based on existing literature.

## Prevalence and Clinical Implications

The co-occurrence of AUD and MDD is common, with studies showing a high comorbidity rate.^2^ Individuals with AUD are more likely to develop depression, and those with depression may engage in heavy drinking.^3^ Their interaction increases the risk of suicide, relapse and poor physical health. Alcohol use can mask underlying MDD, delaying diagnosis and treatment.

Given this complexity, clinicians must also consider alcohol-induced depression (AID), which can present similarly to MDD but typically resolves with sustained abstinence. Early recognition of AID can help tailor appropriate treatment strategies.

## Challenges in Diagnosis

Accurate diagnosis is crucial in treating individuals with both AUD and MDD. AID improves with alcohol abstinence, while MDD persists.^4^ Differentiating between these requires a thorough assessment of the onset, course and relationship of depressive symptoms to alcohol use (Table 1). Persistent symptoms despite abstinence may indicate MDD.

**Table 1.** Clinical Decision Guide: Distinguishing AID versus MDD and Pharmacologic Considerations.

Section	Key features / Examples
Diagnostic differentiation	
Onset	Gradual (MDD) vs After heavy drinking/withdrawal (AID)
Course	Persistent (MDD) vs Fluctuates with drinking (AID)
Abstinence response	Persists after 1 month abstinence (MDD) vs Resolves with abstinence (AID)
Mood features	Melancholic, persistent (MDD) vs Reactive, variable (AID)
Withdrawal-related symptoms	Not prominent (MDD) vs Insomnia, anxiety, tremors common (AID)
Family history	Depression (MDD) vs SUD (AID)
Pharmacologic treatment	
SSRIs (e.g., sertraline)	First-line for MDD; monitor for akathisia and alcohol interaction
SNRIs (e.g., venlafaxine)	May worsen anxiety or BP; risk of serotonin syndrome
Mirtazapine	Good for sleep/appetite; sedating; weight gain
Bupropion	Avoid in seizure risk or withdrawal; stimulating
Imipramine (TCA)	Effective but lethal in overdose; avoid in suicidal patients
Naltrexone	First-line for AUD; contraindicated in liver failure; avoid opioids
Acamprosate	Promotes abstinence; minimal interactions
Disulfiram	Adherence-dependent; avoid alcohol strictly

Sources: DSM-5-TR; McHugh & Weiss, 2019; Hunt et al., 2020; Yoon, 2018; Bahji et al., 2024.

## Integrated Treatment Approaches

1. **Pharmacotherapy**: Managing co-occurring AUD and MDD often involves a combination of pharmacological treatments to target both disorders simultaneously. The choice of medications must be made carefully to avoid interactions and side effects that could exacerbate either condition.
  - **Antidepressants**: Selective serotonin reuptake inhibitors (SSRIs), such as sertraline and fluoxetine, are commonly used to treat MDD in individuals with AUD.^5^ These medications are generally well-tolerated, but caution is needed, as SSRIs can occasionally induce akathisia, a condition that may mimic anxiety or alcohol cravings. Tricyclic antidepressants (TCAs) like imipramine may be effective in severe cases but carry overdose risk, particularly in suicidal patients, and must be used cautiously.
  - **Medications for AUD**: First-line medications for AUD include naltrexone and acamprosate, both of which help reduce alcohol cravings and promote abstinence.^6^ Disulfiram can be considered in selected patients but requires close monitoring. For individuals with concurrent depression, combining antidepressants with AUD medications can enhance overall treatment effectiveness.
2. **Psychotherapy**: Psychotherapeutic interventions are integral in managing co-occurring AUD and MDD. Evidence-based therapies such as cognitive-behavioural therapy (CBT) and motivational interviewing (MI) have demonstrated efficacy for both alcohol use and depressive symptoms.^7^
  - CBT focuses on identifying and modifying negative thought patterns and behaviours that contribute to both alcohol use and depression. CBT reduces cravings, improves coping and alleviates depression.
  - MI helps increase a patient's motivation for change by exploring ambivalence towards treatment. MI is particularly helpful for individuals who may not be ready to fully engage in treatment or abstain from alcohol.
  - Behavioural activation (BA) promotes meaningful activity to counter anhedonia and improve coping with alcohol cravings.
3. **Integrated care models**: The best outcomes for individuals with co-occurring AUD and MDD come from integrated treatment models, where both disorders are addressed simultaneously.^8^ These models involve coordinated pharmacotherapy and psychotherapy, with ongoing follow-up to ensure that both conditions are adequately treated. Integrated care should be tailored to the patient's specific needs, with treatment adjusted based on their progress in both areas.

## Case Example: Integrated Treatment Success

A 33-year-old woman with a history of AUD and MDD presented for assessment. Her history included childhood trauma and family history of SUD and MDD. She had previously tried naltrexone for AUD without success but found acamprosate helpful in reducing alcohol use. Her depression was managed with sertraline and buspirone. Despite successfully reducing her alcohol consumption with acamprosate, she struggled with low mood, anhedonia and social withdrawal. Given her persistent depressive symptoms, an integrated treatment approach was recommended, combining sertraline (antidepressant), naltrexone (AUD medication) and individual psychotherapy (CBT). After 12 weeks, the patient showed significant improvement in both mood and alcohol use. She reported fewer cravings, better sleep and sustained abstinence with regular therapy and social support. This case demonstrates the importance of an integrated treatment approach that targets both alcohol use and depressive symptoms. By addressing both conditions simultaneously, the patient achieved better outcomes than with a sequential treatment approach. Her depressive symptoms persisted well beyond a month of reduced alcohol use, were independent of withdrawal periods and included melancholic features (e.g., early morning awakening, psychomotor slowing), supporting a diagnosis of primary MDD rather than AID.

## Clinical Pearl

The guidance in this article is derived from a narrative synthesis of high-quality studies, published clinical guidelines and recent systematic reviews or meta-analyses where available. Our aim is to equip clinicians with pragmatic tools for decision-making, recognizing the nuanced and often overlapping features of co-occurring AUD and MDD in real-world settings.

1. **Differentiating depression in AUD patients**: Clinicians must carefully assess the type of depression in patients with AUD. AID tends to improve with alcohol cessation, while MDD persists despite alcohol reduction or abstinence. Persistent symptoms despite abstinence may indicate MDD. In cases of AID, abstinence often leads to symptom resolution. Antidepressant treatment is typically reserved for patients whose symptoms persist beyond four weeks of sobriety, suggesting an independent depressive disorder such as MDD.
2. **Choosing medications**: When selecting antidepressants, consider interactions with AUD medications and the patient's history of side effects. SSRIs are commonly used, but TCAs can be effective in severe cases. Medication choice should reflect history, side effects and response.^9^
3. **Incorporating psychotherapy**: CBT and MI should be integrated into treatment plans for individuals with co-occurring AUD and MDD. These therapies improve both alcohol use and depressive symptoms. BA also helps address anhedonia and cravings.
4. **Monitoring and follow-up**: Regular follow-up is essential in managing co-occurring AUD and MDD. Clinicians should monitor for medication adherence, assess treatment progress and provide ongoing support through psychotherapy and social engagement.
